# A Rapid Appraisal of Factors Influencing Praziquantel Treatment Compliance in Two Communities Endemic for Schistosomiasis in Côte d’Ivoire

**DOI:** 10.3390/tropicalmed3020069

**Published:** 2018-06-19

**Authors:** Jean T. Coulibaly, Mamadou Ouattara, Beatrice Barda, Jürg Utzinger, Eliézer K. N’Goran, Jennifer Keiser

**Affiliations:** 1 Swiss Tropical and Public Health Institute, P.O. Box, CH-4002 Basel, Switzerland; beatrice.barda@gmail.com (B.B.); juerg.utzinger@swisstph.ch (J.U.); jennifer.keiser@swisstph.ch (J.K.); 2 University of Basel, P.O. Box, CH-4001 Basel, Switzerland; 3 Unité de Formation et de Recherche Biosciences, Université Félix Houphouët-Boigny, 01 BP 770, Abidjan 01, Côte d’Ivoire; mamadou_ouatt@yahoo.fr (M.O.); eliezerngoran@yahoo.fr (E.K.N.); 4 Centre Suisse de Recherches Scientifiques en Côte d’Ivoire, 01 BP 1303, Abidjan 01, Côte d’Ivoire

**Keywords:** Côte d’Ivoire, coverage rate, praziquantel, preventive chemotherapy, *Schistosoma haematobium*, *Schistosoma mansoni*

## Abstract

Over the past decade, a significant reduction in the prevalence of schistosomiasis has been achieved, partially explained by the large-scale administration of praziquantel. Yet, the burden of schistosomiasis remains considerable, and factors influencing intervention coverage are important. This study aimed to deepen the understanding of low treatment coverage rates observed in two schistosomiasis-endemic villages in Côte d’Ivoire. The research was conducted in August 2015, in Moronou and Bigouin, two villages of Côte d’Ivoire that are endemic for *Schistosoma haematobium* and *S. mansoni*, respectively. After completion of a clinical trial, standard praziquantel treatment (single 40 mg/kg oral dose) was offered to all village inhabitants by community health workers using a house-to-house approach. Factors influencing treatment coverage were determined by a questionnaire survey, randomly selecting 405 individuals. The overall treatment coverage rate was only 47.6% (2730/5733) with considerable intervillage heterogeneity (27.7% in Bigouin (302/1091) versus 52.3% in Moronou (2428/4642)). Among the 200 individuals interviewed in Moronou, 50.0% were administered praziquantel, while only 19.5% of the 205 individuals interviewed in Bigouin received praziquantel. The main reasons for low treatment coverage were work-related (agricultural activities), the bitter taste of praziquantel and previous experiences with adverse events. The most suitable period for treatment campaigns was reported to be the dry season. More than three-quarter of the interviewees who had taken praziquantel (overall, 116/140; Moronou, 84/100; Bigouin, 32/40) declared that they would not participate in future treatments (*p* < 0.001). In order to enhance praziquantel treatment coverage, careful consideration should be given to attitudes and practices, such as prior or perceived adverse events and taste of praziquantel, and appropriate timing, harmonized with agricultural activities. Without such understanding, breaking the transmission of schistosomiasis remains a distant goal.

## 1. Introduction

Schistosomiasis is a widespread neglected tropical disease with a considerable public health impact. Indeed, 779 million people are at risk of schistosomiasis, more than 250 million people are infected with blood flukes of the genus *Schistosoma*, and the global burden in 2016 was estimated at 1.864 million disability-adjusted life years [1,2,3]. More than 90% of schistosome infections are concentrated in Africa [1,4]. There is growing evidence of a positive relationship between schistosome infections and subtle morbidity, including educational, learning and memory deficits [5]. The global strategy is morbidity control, emphasizing periodic administration of praziquantel to at-risk populations without prior diagnosis. This strategy is phrased ‘preventive chemotherapy’ with the declared aim to achieve at least 75% of treatment coverage among school-aged children in schistosome-endemic areas [6]. Efforts are underway to eliminate schistosomiasis as a public health problem by 2025 [7,8,9]. In order to reach this ambitious goal, treatment with praziquantel needs to be administered repetitively with high coverage, in concert with ancillary measures, such as water, sanitation and hygiene (WASH), information, education and communication (IEC) and snail control [10,11,12,13,14,15].

In Côte d’Ivoire, schistosomiasis control efforts have been intensified since 2006, yet were challenged during periods of social unrest, armed conflict and war [16,17]. Starting in 2013, with financial support from the Schistosomiasis Control Initiative (SCI) and other donors, regular large-scale administration of praziquantel is under way, including sporadic assessment of treatment coverage. However, factors influencing coverage rates and reasons for noncompliance have yet to be systematically evaluated in Côte d’Ivoire.

In general, investigations that systematically evaluate treatment coverage and compliance of the community are scarce [18,19,20]. Recent studies carried out in Uganda [18] and Zanzibar [19] determined underlying factors responsible for low treatment compliance. The main reasons identified by Knopp and colleagues for communities in Zanzibar not receiving or taking praziquantel were: absence during drug distribution, no drug distributor reached the household, fear of adverse events, pregnancy, breastfeeding or feeling healthy [19]. Moreover, lack of motivation or professional expertise of drug distributors and limited information of the target population were associated with low compliance of local communities with preventive chemotherapy [21,22]. Chami and colleagues identified additional reasons for low compliance: They found that individuals of low socioeconomic status, religious minorities and small tribes showed particularly low compliance rates with preventive chemotherapy [18].

Within the frame of a randomized controlled trial conducted in two villages of central and western Côte d’Ivoire that assessed the efficacy and safety of potential new treatments (i.e., Synriam^®^, moxidectin and Synriam^®^–praziquantel) against schistosomiasis [12], there was an opportunity to determine praziquantel treatment coverage rates. Indeed, after completion of the clinical trial, a single 40 mg/kg oral dose of praziquantel was offered to the communities. Low compliance was observed, and hence, a questionnaire survey was conducted to identify reasons that might explain lack of compliance. Here we present findings on attitudes and practices towards treatment of schistosomiasis in the two study communities. Results and lessons might help to overcome current challenges in order to achieve effective control and elimination of schistosomiasisin Côte d’Ivoire and other countries.

## 2. Material and Methods

### 2.1. Ethical Approval and Study Setting

The study was conducted in August 2015 during the rainy season, following completion of a clinical trial assessing the efficacy and safety of Synriam^®^ and moxidectin [12] against urogenital and intestinal schistosomiasis in 256 school-aged children and adolescents (age: 12–17 years) in the villages of Moronou (central Côte d’Ivoire) and Bigouin (western Côte d’Ivoire). As described elsewhere, Moronou and Bigouin are highly endemic for *Schistosoma haematobium* and *S. mansoni*, respectively [12]. The clinical trial was approved by the Ethics Committee of Northwestern and Central Switzerland (EKNZ; reference no. 15/01) and the Comité National d’Éthique et de la Recherche (CNER; reference no. 026, approval date: 16 June 2015) of the Ministry of Health in Côte d’Ivoire. According to the last national census carried out in 2014, there were 4642 and 1091 inhabitants in Moronou and Bigouin, respectively [23]. Informed oral consent was obtained from all participants for the questionnaire survey.

### 2.2. Sample Size Calculation

The sample size was assessed as recommended by Lemeshow et al. [24]. Briefly, we assumed that 90% (*P*) of the individuals approached would answer the questionnaire with a relative precision (*d*) of 5% and a level of confidence of 95% (Z = 1.96). The minimum sample size for each setting was 138 individuals, calculated as follows: *n* = *Z*^2^_1−α/2_*P*(1 − *P*)/*d*^2^.

Allowing for 30% of absence during the door-to-door visit, approximately 200 individuals (school-aged children, 6–15 years and adolescents/adults, ≥16 years) were randomly selected in each village with equal numbers of treated and nontreated individuals during the door-to-door treatment (as described below).

### 2.3. Praziquantel Treatment

**Figure 1:**
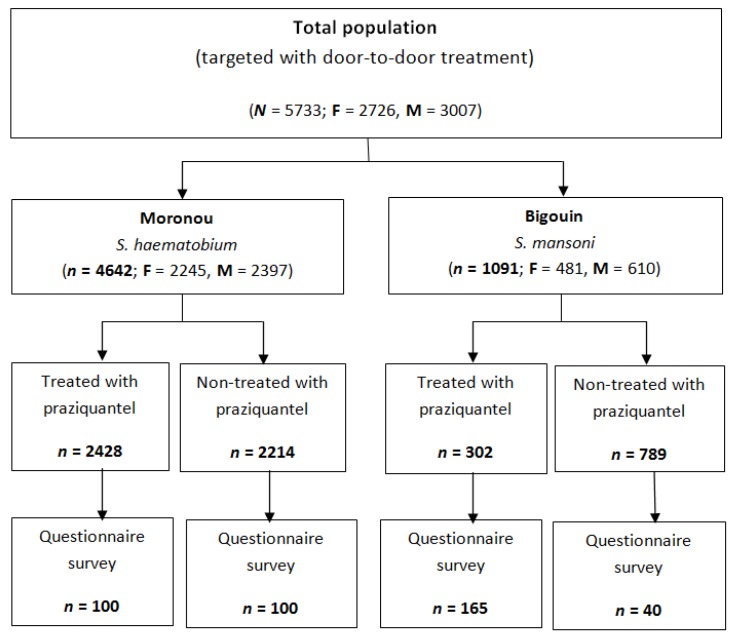
Study profile.

Praziquantel tablets (Cesol^®^ 600 mg) were offered free of charge to both communities following the completion of a clinical trial (Figure 1). Communities were informed about the treatment days, and the objectives, procedures and potential risks and benefits were explained. Village chiefs and community leaders conducted an information meeting and, subsequently, the information was passed on to the entire population. In both villages, a door-to-door approach was employed to administer praziquantel over two consecutive days. In each village, a community health worker (CHW) was recruited per neighborhood (six in Moronou and four in Bigouin). The CHWs were trained to treat community members using a dose pole [25,26]. Villagers were invited to swallow the drug in front of the CHWs. Related data, including name, sex, age and number of tablets administered, were recorded in a register used by the national schistosomiasis control programme.

### 2.4. Questionnaire Assessing Factors Influencing Treatment Compliance

A structured questionnaire was created, including data on demographics, main activities of the participants, whether they had accepted the recent praziquantel treatment and factors that influenced treatment compliance ( Supplementary file 1). We also gathered suggestions on how to improve the distribution and administration of treatment in order to increase compliance. The questionnaire was pretested in Abidjan, the economic capital of Côte d’Ivoire, among 15 randomly selected children aged 9–12 years. CHWs were trained to implement the questionnaire. They were fluent in the local language (i.e., Baoulé in Moronou and Yacouba in Bigouin). Translation of the questionnaire into local languages was done for each study location. The questionnaire was implemented over 7 days in each village, after the house-to-house treatment.

### 2.5. Statistical Analysis

Data were double entered into a database using EpiInfo version 3.5.1. (Centers for Disease Control and Prevention, Atlanta, GA, USA). Data analysis was done using STATA version 12.1 (StataCorp., College Station, TX, USA). Qualitative (e.g., reasons for noncompliance) and quantitative data (e.g., age and sex) were expressed as proportions and means, respectively. Proportions were compared using Pearson’s chi-squared test (χ^2^). A *p*-value less than or equal to 0.05 was considered as statistically significant.

## 3. Results

### 3.1. Treatment Compliance

Table 1 shows the praziquantel treatment coverage rate in Moronou and Bigouin. The overall coverage rate was 47.6% (2730/5733) with considerable difference between the two villages (27.7% in Bigouin (302/1091) versus 52.3% in Moronou (2428/4642)). While males reported significantly higher treatment compliance in Bigouin (χ^2^ = 19.96, *p* < 0.001), no sex difference was observed in Moronou (χ^2^ = 0.16, *p* = 0.104).

### 3.2. Factors Associated with Coverage of Praziquantel Treatment

Table 2 summarizes sex, age and occupation of the participants interviewed. The factors influencing treatment compliance and suggestions on how treatment programmes could be improved are presented in Table 3. In both settings we interviewed more males than females (248 versus 157) with a statistically-significant difference observed in Bigouin (145 males versus 60 females; χ^2^ = 23.95, *p* < 0.001). The main activity of the participants was farming with cash crops, particularly coffee, cacao and hevea (53.5% in Moronou and 48.8% in Bigouin), followed by vegetables and rice (20.5% and 19.5%, respectively).

Half of the interviewees in Moronou (*n* = 100, 50%) and four out of five interviewees in Bigouin (*n* = 165, 80.5%) did not take praziquantel during the treatment campaign. Farming activities were stated as the main reason for not participating in the treatment (70%, 70/100 in Moronou; 89.7%, 148/165 in Bigouin). Other factors were adverse events experienced in previous treatment campaigns (25.0%, 25/100 in Moronou) and a lack of treatment need (7.9%, 13/165 in Bigouin). Additionally, 26.0% (26/100) of the participants in Moronou and 37.5% (15/40) in Bigouin complained about the bitter taste of praziquantel as a reason for refusing treatment. Among the participants who accepted the treatment, 62.0% (62/100) in Moronou and 52.5% (21/40) in Bigouin complained about adverse events.

We were also interested in learning whether interviewees would accept another round of praziquantel treatment and if so, whether they could give us some indications on the most appropriate approach and timing for treatment. In both villages, among the individuals who were willing to accept another treatment, three-quarters (overall 116/140; Moronou 84/100; Bigouin 32/40) declared that they would not participate in a future treatment round (*p* < 0.001). Study participants recorded that in both villages the most suitable period of the year for treatment campaigns would be the dry season. There was no preferable time during the course of the day for the treatment. When asked about the most suitable approach for the treatment delivery, in Moronou, two-third (68.5%, 137/200) suggested a house-to-house approach as used in the current study (*p* < 0.001). There was no specific preference in Bigouin (*p* = 0.200).

## 4. Discussion

Schistosomiasis is a neglected tropical disease that remains of considerable public health relevance [3,27]. The cornerstone of schistosomiasis control is preventive chemotherapy, emphasising periodic treatment of school-aged children with the declared aim to treat at least 75% of this age group in schistosomiasis-endemic areas [7,8]. In view of new aspirations to eliminate schistosomiasis as a public health problem and to break transmission, a range of activities, including broadening of preventive chemotherapy along with WASH, IEC and snail control, are required [15,28,29]. Expanding preventive chemotherapy programmes to the adult population calls for investigations of low treatment coverage and compliance issues observed in several endemic areas that might undermine the success of preventive chemotherapy [30].

Assessment of treatment compliance has been done mainly for mass drug administration against lymphatic filariasis [21,31]. Only a few studies focused on schistosomiasis and intestinal helminthiases [19,32,33]. Moreover, in Côte d’Ivoire, the factors influencing praziquantel coverage rates have yet to be investigated.

We pursued a cross-sectional questionnaire survey to study factors that might explain low treatment coverage in two selected villages in which a small clinical trial had been conducted before the current study [12]. We employed a door-to-door approach with previously trained CHWs. While the treatment compliance in Bigouin was very low (27.7%), it was considerably higher in Moronou (52.3%). The main reason for not having received or taken praziquantel was farming activities at the time of drug administration. Additional reasons were experience with adverse events from previous treatments and the bitter taste of praziquantel. Although the bitter taste of praziquantel and adverse events are well acknowledged in the literature [34,35,36,37], to our knowledge, studies evaluating the impact of these issues on treatment compliance are scarce [35].

Previous studies assessing praziquantel treatment coverage reported varying compliance rates and a host of factors influencing coverage. It might be worth highlighting that most of the data reported thus far are based on systematic reviews, but only few have considered coverage as a study objective and assessed it thoroughly using a questionnaire approach [19,38]. However, a standard questionnaire used by the research teams to evaluate the factors influencing the treatment coverage is lacking, rendering comparison from one location to another difficult [39,40,41].

Advice has been given by the interviewed population to preferably implement preventive chemotherapy in the dry season when farming activities are significantly reduced. Note that in Côte d’Ivoire, as in other African countries, the rainy season is a period of intense farming activities. In that period, a high proportion of the population is staying in small temporary hamlets, often inaccessible to CHWs. It is clear that, while schoolchildren can be easily reached through the education system [42], when extending the treatment to the entire community, socio–cultural issues, accessibility and main occupational activities should be considered through a participatory approach [21]. Another factor to be taken into account might be the duration of the intervention. Krentelet al. reported that in some settings, the scheduled time for drug distribution was not sufficient to cover the entire target population. Hence, one option would be to increase time dedicated to preventive chemotherapy and to increase the number of CHWs distributing praziquantel. Yet, this suggestion was not made by the people interviewed in the current investigation.

Our study has several limitations. First, the research was restricted to two rural settings of central and western Côte d’Ivoire. In previous studies, distinctively different treatment compliance rates were observed when comparing rural and urban settings; usually compliance in urban areas is lower [21,31]. This was attributed mostly to a high proportion of migrants and mobile population, private health institutions that discourage people from participating in preventive chemotherapy and lack of a specific urban health strategy. Second, the treatment approach used by CHWs might differ based on the size of the location. We found that in Moronou, which is approximately three times larger than Bigouin, the population prefers a house-to-house approach. On the other hand, in Bigouin, no preferable drug distribution emerged, as revealed by our questionnaire survey. As expected, treatment needs by different populations vary and further investigations are required to elucidate this issue. Third, we evaluated the current praziquantel treatment coverage rates shortly after the completion of a small clinical trial in adolescents, assessing the efficacy and safety of different novel treatments and treatment combinations against schistosomiasis, including praziquantel. Of note, during this trial only a few participants from each community experienced adverse events, most of which were mild and transient. Yet, it is conceivable that people experiencing adverse events or complaining of the bitter taste of praziquantel might have influenced the willingness of the community members to participate in the current mass treatment.

Only a few studies determined the impact of repeated preventive chemotherapy on treatment compliance with praziquantel. In settings that underwent several years of mass drug administration, such as in Zanzibar [19], Knopp et al. speculated on ‘treatment fatigue’ of the population being only marginally infected, but repeatedly given medications. Similarly, in our study, we found that most of the interviewees denied a subsequent treatment. This is an important issue, which needs careful attention by disease control programme managers, since predictive models taking into account prevalence and treatment coverage have shown that to reach elimination with mass drug administration, at least 10 years of regular treatment with high treatment coverage (>70%) is required [43]. In settings that aim at breaking transmission, integrated control approaches are warranted, complementing preventive chemotherapy with WASH, IEC and snail control [44,45,46]. Such integrated control programmes require long-term commitment [47].

In conclusion, with schistosomiasis morbidity control progressively moving towards elimination, and hence broadening preventive chemotherapy from school-aged children to include preschool-aged children and adults, efforts should be made to regularly assess treatment coverage rates. Since morbidity of schistosomiasis is often subtle and individuals might not feel sick, sensitization prior to treatment should be carried out to diminish the reluctance of the population towards the treatment caused by adverse events and the bitter taste of praziquantel. Particular attention should be given to idiosyncrasies of endemic areas by taking into account timing and the mix of the intervention.

## Figures and Tables

**Table 1:** Praziquantel treatment coverage in Moronou and Bigouin, endemic respectively for *Schistosoma haematobium* and *S. mansoni*, in Côte d’Ivoire. The denominators for the treatment coverage rate were derived from the 2014 national census.

Characteristic	Total Population	χ^2^	*p*	No. of People Treated	Treatment Coverage %	χ^2^	*p*
Bigouin							
Male	610			212	34.8		
Female	481	10.19	0.001	90	18.7	19.96	<0.001
Total	1091			302	27.7		
Moronou							
Male	2397			1266	52.8		
Female	2245			1162	51.8	0.16	0.104
Total	4642	3.32	0.069	2428	52.3		
**Total**	5733			2730	47.6	85.39	<0.001

**Table 2:** Characteristics of study participants.

Participant Characteristics	Moronou (*n* = 200)			Bigouin (*n* = 205)			Total (*N* = 405)		
	*n* (%)	χ^2^	*p*	*n* (%)	χ^2^	*p*	*n* (%)	χ^2^	*p*
**Sex**									
Female	97 (48.5)			60 (29.3)			157 (38.8)		
Male	103 (51.5)	0.12	0.729	145 (70.7)	23.95	<0.001	248 (61.2)	13.71	<0.001
**Average Age (SD)**									
6–15 years	11.9 (2.5)			11.3 (2.2)			11.6 (2.4)		
≥16 years	36.6 (14.0)			40.1 (14.3)			38.4 (14.3)		
**Occupation**									
Cash crops (cocoa, coffee, hevea) (1)	107 (53.5)			100 (48.8)			207 (51.1)		
Vegetable and rice crops (2)	41 (20.5)			40 (19.5)			81 (20.0)		
Combined activities (1 & 2)	18 (9.0)			50 (24.4)			68 (16.8)		
Other activity (trade, civil servant, etc.)	34 (17.0)	66.84	<0.001	15 (7.3)	57.15	<0.001	49 (12.1)	109.87	<0.001

**Table 3:** Factors associated with praziquantel treatment coverage rates in Moronou and Bigouin, Côte d’Ivoire.

Associated Factor	Moronou (*n* = 200)			Bigouin (*n* = 205)			Total (*N* = 405)		
	*n* (%)	χ^2^	*p*	*n* (%)	χ^2^	*p*	*n* (%)	χ^2^	*p*
**Accepted Praziquantel Treatment (MDA)**									
Yes	100 (50.0)			40 (19.5)			140 (34.6)		
No	100 (50.0)	n.a.	n.a.	165 (80.5)	53.00	<0.001	265 (65.4)	26.00	<0.001
**Reason for Denial of Treatment**	***n* = 100**			***n* = 165**			***n* = 265**		
Not sick	4 (4.0)			13 (7.9)			17 (6.4)		
Adverse events of previous treatment	25 (25.0)			3 (1.8)			28 (10.6)		
Busy with field activities	70 (70.0)			148 (89.7)			218 (82.3)		
Not informed about the treatment	1 (1.0)	87.42	<0.001	1 (0.6)	237.48	<0.001	2 (0.7)	306.93	<0.001
**Reason of Treatment’s Acceptance**	***n* = 100**			***n* = 40**			***n* = 140**		
Sick	7 (7.0)			2 (5.0)			9 (6.4)		
Knowledge on schistosomiasis/disease	68 (68.0)			11 (27.5)			79 (56.4)		
Emulation	25 (25.0)	42.57	<0.001	27 (67.5)	17.86	<0.001	52 (37.1)	43.33	<0.001
**What you did not like with the drug?**	***n* = 100**			***n* = 40**			***n* = 140**		
The taste	26 (26.0)			15 (37.5)			41 (29.3)		
The size of the tablet	12 (12.0)			4 (10.0)			16 (11.4)		
Adverse events	62 (62.0)	28.69	<0.001	21 (52.5)	8.98	<0.008	63 (45.0)	22.06	<0.001
**Impact of the drug on your wellbeing**	***n* = 100**			***n* = 40**			***n* = 140**		
No effect	15 (15.0)			4 (10.0)			19 (13.6)		
Improvement	85 (85.0)	34.55	<0.001	36 (90.0)	18.37	<0.001	121 (86.4)	52.65	<0.001
**Could you accept a new treatment?**	***n* = 100**			***n* = 40**			***n* = 140**		
Yes	16 (16.0)			8 (20.0)			24 (9.2)		
No	84 (84.0)	32.50	<0.001	32 (80.0)	10.00	<0.002	116 (44.4)	48.61	<0.001
**Appropriate period for the treatment**	***n* = 200**			***n* = 205**			***n* = 405**		
Rainy season	2 (1.0)			7 (3.4)			9 (2.2)		
Dry season	198 (99.0)	143.35	<0.001	198 (96.6)	131.30	<0.001	396 (97.8)	274.37	<0.001
**Appropriate time for the treatment**	***n* = 200**			***n* = 205**			***n* = 405**		
Early in the morning	15 (7.5)			2 (1.0)			17 (4.2)		
In the evening	14 (7.0)			26 (12.7)			40 (9.9)		
At any time, if I am informed	171 (85.5)	164.12	<0.001	177 (86.3)	179.78	<0.001	348 (85.9)	340.58	<0.001
**What is the best place to facilitate treatment?**	***n* = 200**			***n* = 205**			***n* = 405**		
Central part of the village	16 (8.0)			78 (38.0)			94 (23.2)		
House-to-house distribution	137 (68.5)			55 (26.8)			192 (47.4)		
Health centre	47 (23.5)	84.39	<0.001	72 (35.1)	3.19	0.200	119 (29.4)	27.90	<0.001

## Supplementary Materials

Available online http://www.mdpi.com/2414-6366/3/2/69/s1.
