# Supplementary material for: A Rapid Appraisal of Factors Influencing Praziquantel Treatment Compliance in Two Communities Endemic for Schistosomiasis in Côte d’Ivoire
Source: Trop Med Infect Dis. 2018 Jun 19;3(2):69. doi: 10.3390/tropicalmed3020069 (PMC6073597; doi:10.3390/tropicalmed3020069)
Supplement: Supplementary file 1 [file tropicalmed-03-00069-s001.pdf]

No. ID

Questionnaire for praziquantel mass treatment coverage evaluation in rural areas in Côte d'Ivoire

(Note: this questionnaire targets school-aged children (6-15 years) and adults (≥16 years))

Interviewer name: …………………………………………………………………..

Health district: …………………………………………………………………..

Location: …………………………………………………………………..

PARTICIPANT IDENTIFICATION

First name: …………………………….. Family name: ………………………………………………………..

Age: …………….. (years)

Sex: M ☐ F ☐

PARTICIPANT MAIN ACTIVITY

What is your main activity? (only for adults, ≥16 years)

Cocoa growing ☐ Coffee growing ☐ Rubber growing ☐ Vegetable crops ☐

Rice culture ☐ Trade ☐ Others ☐(Specify, …………………………………………….)

What is the main activity of your parents? (Only for school-aged children, 6-15 years)

Cocoa growing ☐ Coffee growing ☐ Rubber growing ☐ Vegetable crops ☐

Rice culture ☐ Trade ☐ Others ☐(specify, …………………………………………….)

ADHERENCE TO MASS TREATMENT

Have you recently received treatment for schistosomiasis?

Yes ☐ No ☐

If so, why did you agree to take the medicine?

I felt sick ☐ I wanted to do like the others ☐ I know that schistosomiasis is bad for my health ☐

Other ☐ (specify,………………………………………………………………………………..)

What did you not like during the treatment?

The taste of the medicine ☐ The size of the tablets ☐ Feeling after treatment ☐

Other ☐ specify, ………………………………………………………………………………)

How did you feel a week after treatment?

Bad ☐ Well ☐ Very well ☐ Nothing was changed ☐

No. ID

If you have to take once more the medicine will you do it?

Yes ☐ No ☐

If no, why did you not agree to be treated once more?

I did not feel sick ☐ I once took the medicine and got trouble ☐ I was concerned about my field work ☐ I have not been informed ☐ My parents refused to take it ☐ Other (specify ……………………………………………………………………………………………….)

When do you think is the right period for mass treatment?

Dry season ☐ Rain season ☐ Mornings ☐ Nights ☐

At any time if we are well informed ☐ Other ☐ (specify, ……………………………………….)

What is the best place for you to do the treatment?

Public place ☐ Health centre ☐ Door-to-door ☐ Other ☐
(specify, ……………………………………………………………………………………………….)
